# Tissue Xpert® MTB/RIF Assay in Peritoneal Tuberculosis: To be (Done) or Not to be (Done)

**DOI:** 10.7759/cureus.5009

**Published:** 2019-06-26

**Authors:** Amol S Dahale, Amarender S Puri, Ajay Kumar, Ashok Dalal, Anil Agarwal, Sanjeev Sachdeva

**Affiliations:** 1 Gastroenterology, G B Pant Institute of Postgraduate Medical Education and Research, New Delhi, IND; 2 Gastrointestinal Surgery, G B Pant Institute of Postgraduate Medical Education and Research, New Delhi, IND

**Keywords:** abdominal tuberculosis, peritonitis, diagnostic laparoscopy, gene xpert

## Abstract

Introduction

Peritoneal tuberculosis (PTB) is a paucibacillary disease with poor mycobacterial yield in ascitic fluid. The Xpert® MTB/RIF assay (Gene Xpert) is a new tool for the diagnosis of tuberculosis (TB) and has not yet been studied on peritoneal tissue. The present study aimed to investigate the yield of the Xpert® MTB/RIF assay on peritoneal tissue obtained at peritoneoscopy.

Methods

This is a retrospective study and the data were collected from hospital records. The patients who underwent peritoneoscopy along with Xpert® MTB/RIF assay on peritoneal tissue were included in this study. Those with proven PTB were considered as cases while those with other diagnoses as controls. Using the reference standard of TB diagnosis, sensitivity, specificity, and accuracy of Xpert® MTB/RIF assay were calculated.

Results

Total of 36 patients was analyzed in this study: 28 as cases and eight as controls. Peritoneoscopy was carried out for diagnosis and biopsy. Histopathology in cases revealed caseating granulomas in 16 while 11 had non-caseating granulomas. Nine patients showed acid-fast bacillus positivity on peritoneal tissue. The most common finding on peritoneoscopy was tubercles with adhesions (n = 14, 50%), followed by tubercles only (n = 12, 42.9%). Xpert® MTB/RIF assay was positive in 17 (60.7%) patients with a sensitivity of 60.71%, specificity of 100%, and an accuracy of 69.44%. Two patients expressed rifampicin resistance.

Conclusion

Xpert® MTB/RIF assay on peritoneal tissue has fair sensitivity and excellent specificity. The multidrug resistance and the ability to provide results rapidly make it clinically useful.

## Introduction

Peritoneal tuberculosis (PTB) forms a significant proportion of abdominal tuberculosis population [1]. Often, diagnosis is difficult in view of nonspecific symptoms and paucibacillary nature of the disease [2-3]. Ascitic fluid adenosine deaminase (ADA) can be used for the diagnosis as it has excellent sensitivity and specificity [4-6]. However, mycobacteriological analysis either by staining or culture remains the gold standard. Moreover, ADA cannot provide information on multidrug resistance (MDR) nature of bacteria [2]. Furthermore, there is also a small but definite chance of false positive results with ADA. Xpert® MTB/RIF (Gene Xpert) assay is the latest addition in the clinician’s armamentarium for the diagnosis of tuberculosis. It uses nested polymerase chain reaction (PCR) technique with automated amplification and detects Mycobacterium tuberculosis and rifampicin resistance gene. The rifampicin resistance gene is an accurate surrogate marker for MDR tuberculosis (TB). Overall, Xpert® MTB/RIF assay has excellent sensitivity and specificity on sputum samples, especially which are acid-fast bacillus (AFB) positive on Zeihl-Neelsen stain. Therefore, Xpert® MTB/RIF is endorsed by the World Health Organization (WHO) for the diagnosis of pulmonary tuberculosis on sputum samples [7]. Nowadays, Xpert® MTB/RIF assay is also increasingly applied to extrapulmonary samples. Recently published meta-analyses highlight this fact. However, studies included in these meta-analyses have few cases of peritoneal TB, and the tests were conducted only on ascitic fluid [8-9]. One study from India has used peritoneal tissue that was obtained by ultrasound-guided biopsy, and hence, was not sufficient to define the exact role of Xpert® MTB/RIF assay on peritoneal tissue [10]. Therefore, the present study aimed to analyze the role of Xpert® MTB/RIF assay in the diagnosis of PTB on peritoneal tissue obtained by peritoneoscopy.

## Materials and methods

This retrospective case-control study deduced the efficacy of Xpert® MTB/RIF assay on peritoneal tissue. We retrieved the data of patients who underwent diagnostic laparoscopy (peritoneoscopy) for undiagnosed ascites due to the possibility of tuberculosis. Hospital records of patients at the Department of Gastroenterology and Gastrointestinal Surgery at G.B. Pant Institute of Postgraduate Medical Education and Research (GIPMER), New Delhi, India from December 2015-December 2017 were screened. All patients aged ≥18 years who underwent peritoneal biopsy along with Xpert® MTB/RIF assay were studied. Patients with incomplete data were excluded. Also, clinical features and parameters such as blood parameters and ascitic fluid analysis were noted. Additionally, reports of the tuberculin test, human immunodeficiency virus (HIV) test and visual findings of peritoneoscopy were recorded in all patients. The peritoneal biopsy was taken from visualized abnormal areas and subjected to histopathology and Xpert® MTB/RIF assay.

Xpert® MTB/RIF assay

The Xpert® MTB/RIF assay is a molecular technique, which uses nested real-time PCR method. The sample for this assay was collected in normal saline and processed within four hours. The Mycobacterium tuberculosis genes (MTB) and genes that confer rifampicin resistance (rpoB) are detected automatically by molecular probes. These are disposable cartridges that have the raw material, i.e., primers as well as molecular probes. It is a robust and rapid method making results available within two hours [7].

Definitions of cases and controls

Those patients who had proven PTB were considered as cases while those with other diagnoses were taken as controls. Definitions of cases and controls are described as follows:

*Cases: *All patients with a definite diagnosis of PTB and those who underwent Xpert® MTB/RIF assay on peritoneal tissue were taken as cases. The definite diagnosis of tuberculosis (reference standard) was considered if any of the following were present [11]:

(1) Histopathology revealed AFB

(2) Caseating granulomas

(3) High clinical suspicion with non-caseating characteristic granuloma (multiple well-defined epithelioid granulomas with Langerhans’s giant cells) and response to antitubercular drugs (ATT)

*Controls: *All other patients who underwent peritoneoscopy but had a different diagnosis and were also subjected to Xpert® MTB/RIF assay testing on peritoneal tissue were taken as controls. All other diagnoses were confirmed by histopathology (for benign and malignant tumors). Endoscopic retrograde pancreatography with leak documentation was utilized for pancreatic ascites diagnosis. Chronic liver disease was diagnosed by ultrasonography, endoscopy, and/or liver biopsy.

Statistical analysis

All continuous data were expressed as mean, standard deviation, and median, while categorical data were expressed as percentages and proportions. Cases and controls parameters (continuous data) were compared using the Mann-Whitney U test and a p-value of less than 0.05 was taken as significant. Sensitivity, specificity, and accuracy were calculated according to the standard formulas. SPSS version 23 was used for statistical analysis.

## Results

A total of 43 patients underwent peritoneoscopy during the study. Tuberculosis was proven in 30 patients. Of these, Xpert® MTB/RIF assay was carried out in 28 patients who were included as cases for the present analysis. Of the remaining non-TB group, Xpert® MTB/RIF assay was conducted in eight patients that served as controls for comparison.

The male to female ratio in cases was 16:12. The mean age of the patients in the case group was 34.60±13 years. Of these, eight had underlying cirrhosis. The most common symptoms were anorexia, weight loss, and abdominal distension (n = 27, 96%), followed by abdominal pain in 26 (93%) patients. Tuberculin skin testing was positive in 19 (67.85%) patients at the cutoff of 10 mm. The ascitic fluid showed high protein (4.65±1.33 gm/dL) with low serum albumin ascitic albumin gradient (SAAG) (0.76±0.38 gm/dL) and high ADA value (70.37±18 IU/L). The blood and ascitic fluid parameters of patients are described in Table 1.

**Table 1:** Blood and ascitic fluid parameters of cases and controls

Parameters	Cases mean (±SD)	Controls mean (±SD)	P value
Age	34.60±13	45.37±18	0.08
Hemoglobin (gm/dL)	9.82±1.90	11.67±1.58	0.04
Platelets (/µL)	2.67±1.46	2.47±1.01	0.61
Leucocyte (cells/ µL)	6710±2118	7300±2673	0.51
Bilirubin (mg/dL)	0.98±1.1	0.71±0.34	0.49
Total protein (gm/dL)	7.20±0.85	6.65±0.69	0.11
Albumin (gm/dL)	3.12±0.87	3.15±0.58	0.94
Ascitic fluid total leucocyte count (cells/ µL)	539±480	334±269	0.26
Ascitic fluid total Protein (gm/dL)	4.65±1.33	4.43±1.04	0.68
Ascitic fluid albumin (gm/dL)	2.23±0.84	1.81±1.06	0.25
Serum albumin ascites albumin gradient (SAAG)	0.76±0.38	0.80±0.62	0.84
Ascitic fluid adenosine deaminase (IU/L)	70.37±18	41.11±43.78	0.007

**Figure 1:**
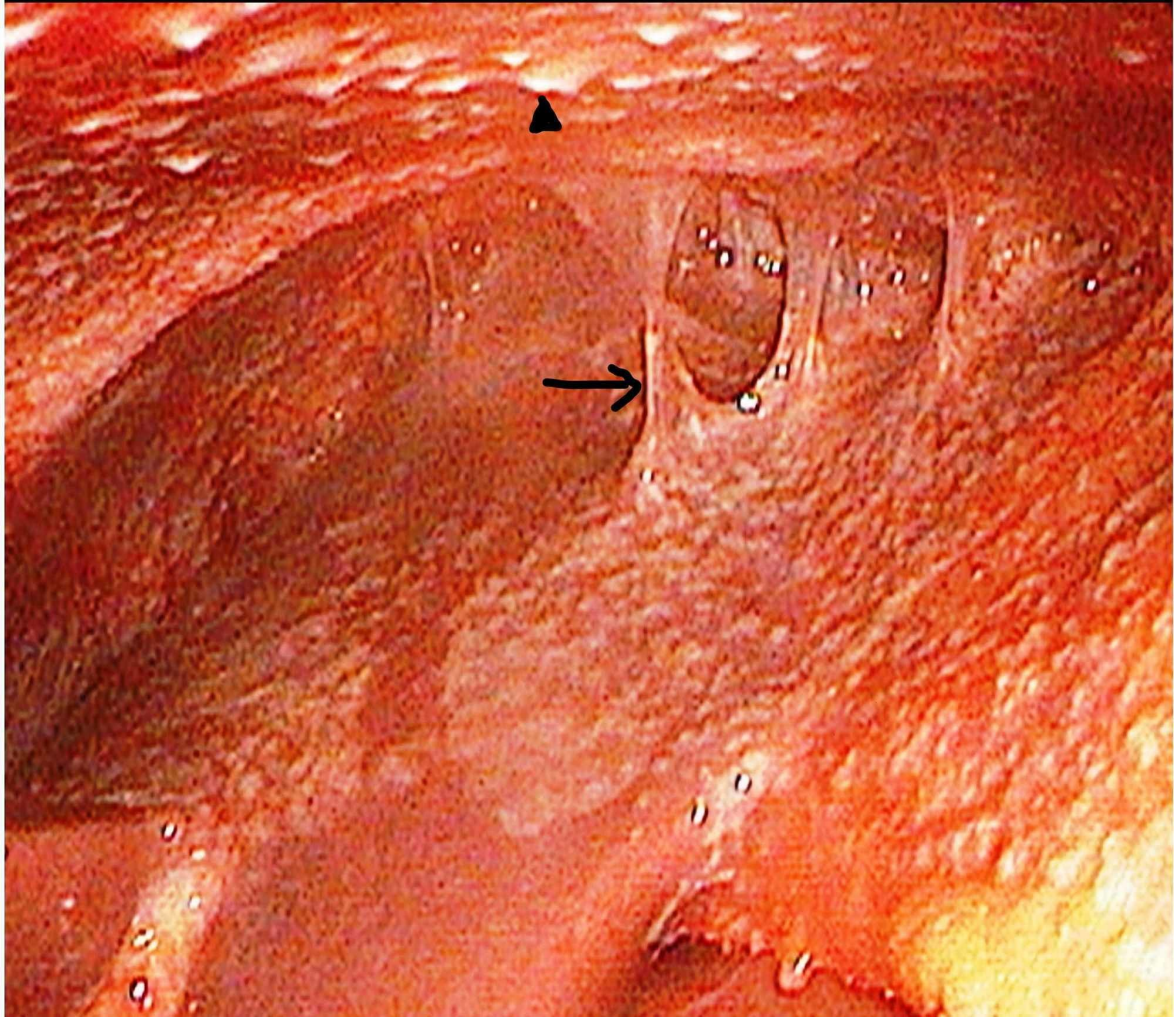
Peritoneoscopy picture showing peritoneal tubercles (arrowhead) with adhesion (arrow)

**Figure 2:**
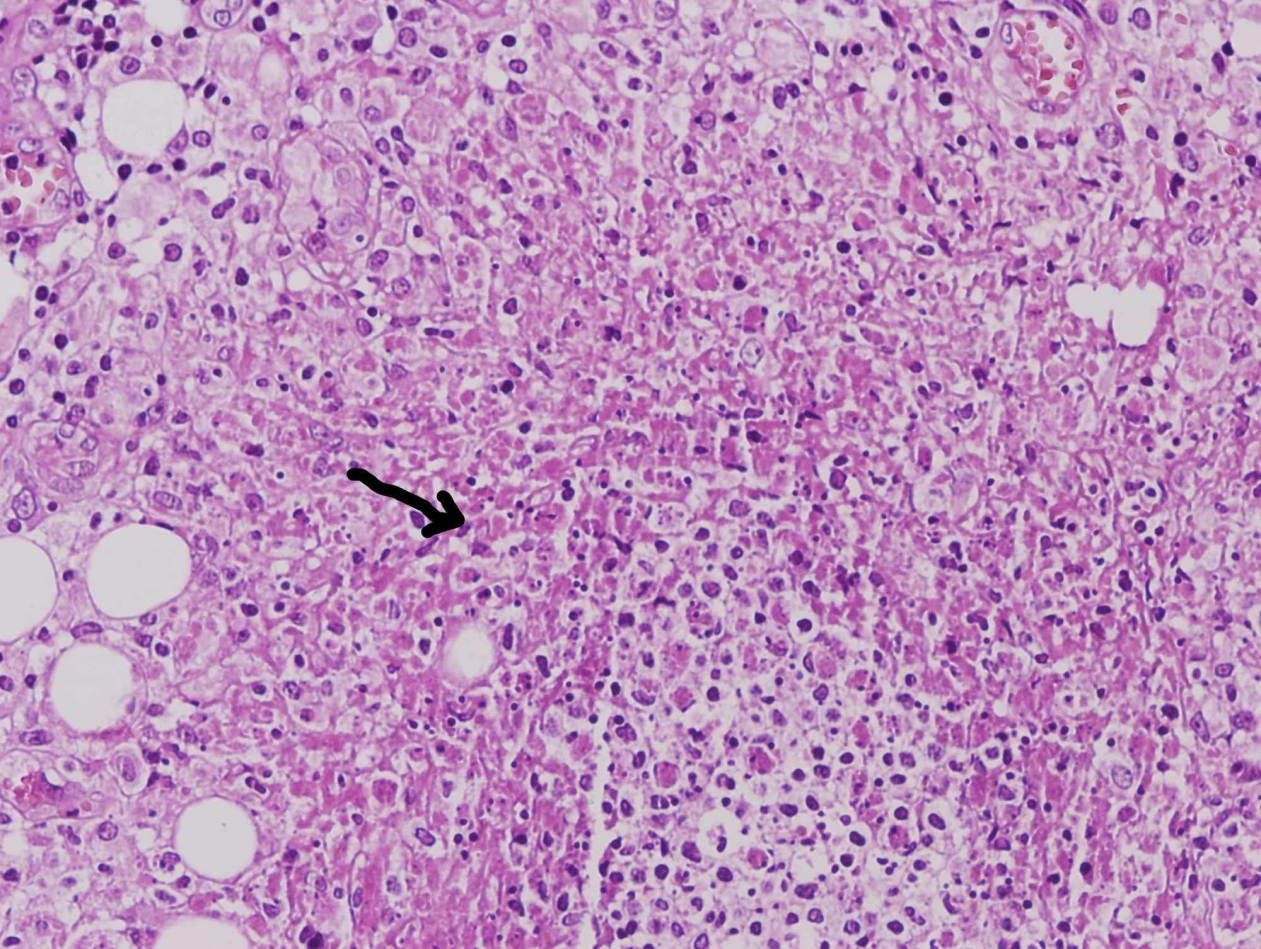
Peritoneal biopsy showing non-caseating granuloma (Hematoxylin and eosin stain) (arrow)

The most common finding on peritoneoscopy was tubercles with adhesions (n = 14, 50%) (Figure 1), followed by tubercles only (n = 12, 42.9%). Peritoneal thickening with tubercles and tubercles with cocoon was seen in one patient each. Histopathology revealed that AFB was positive in nine (32.1%) patients. A total of 16 (57.1%) patients had caseating granulomas, 11 (39.3%) had non-caseating granulomas (Figure 2) while one had non-specific inflammation but was AFB-positive. In addition, Xpert® MTB/RIF assay was found to be positive in 17 (60.7%) (Table 2), and rifampicin resistance was detected in two patients (7.1%).

**Table 2:** Histopathological and Xpert® MTB/RIF assay analysis of cases

Category	Tissue Zeihl-Neelsen stain		Histopathology			Xpert® MTB/RIF assay		Total
	Positive	Negative	Caseating Granuloma	Non-caseating Granuloma	Other	Positive	Negative	
Cases n = 28	9	19	16	11	1	17	11	28

The control group consisted of eight patients. Six patients had malignant ascites as peritoneal biopsy showed malignant deposits. One patient had pancreatic ascites while another had the chronic liver disease only. Peritoneoscopy revealed tubercles in five malignant ascites, while one showed omental caking only. One patient with pancreatic ascites had peritoneal thickening while another patient with chronic liver disease had normal peritoneum. All these patients exhibited negative results for Xpert® MTB/RIF assay.

Sensitivity, specificity, and accuracy of Xpert® MTB/RIF assay

In the present study, Xpert® MTB/RIF assay showed 60.71% (95% confidence interval (CI): 40.58-78.50) a sensitivity, 100% specificity (95% CI: 63.06-100), and accuracy of 69.44% (95% CI: 51.89-83.65).

Treatment

All patients received ATT as per the national guidelines. Those who were Xpert® MTB/RIF assay-positive started ATT on day two, while those who are negatively received ATT after a median duration of seven days.

## Discussion

Tuberculosis is a major health concern in developing countries including India [12]. The contribution of extrapulmonary tuberculosis to the total TB burden is on the rise comprising of PTB, which is difficult to diagnose due to its myriad symptoms and paucibacillary nature [2-3,13]. ADA is a satisfactory non-invasive test which is easily available nowadays and commonly used for the diagnosis of PTB owing to its sensitivity and specificity [4-6]. Despite this, a large number of patients undergo peritoneoscopy for diagnosis due to nonspecific symptoms and similarity to other illnesses [14]. The Xpert® MTB/RIF assay was recently used for the diagnosis of pulmonary TB as well as for the detection of MDR TB and advocated by WHO for sputum testing [7]. Its role in extrapulmonary tuberculosis is being studied increasingly. A few metanalyses have been published, but data on peritoneal tuberculosis are scarce [8-9]. Most studies published on PTB have used ascitic fluid for the Xpert® MTB/RIF assay. The yield of Xpert® MTB/RIF assay in the ascitic fluid was poor from 4%-28% [15-21]. Although this yield may be higher in HIV patients, only limited data are available [19]. The results are as expected due to the paucibacillary nature of the disease and poor yield of smears and cultures in ascitic fluid [2]. Hitherto, only one study has been published on the efficacy of Xpert® MTB/RIF assay on peritoneal tissue in PTB patients, which used ultrasound-guided peritoneal tissue [10]. The current study documented a detailed account of 28 patients with the efficacy of Xpert® MTB/RIF assay on peritoneal tissue acquired by peritoneoscopy.

The present study had promising results. Also, we used robust reference diagnostic criteria that are clinically reliable and relevant. Using these as reference criteria, the sensitivity of Xpert® MTB/RIF assay in the current study was 60.71%, while specificity was 100%. While 100% specificity is not surprising as the Xpert® MTB/RIF assay is highly specific, as compared to a previously published study the assay exhibited high sensitivity in the current study (60.71% vs.19%) [7,10]. This phenomenon could be attributed to the abundant tissue obtained by peritoneoscopy. Gene Xpert also detected MDR TB in two patients (7.14%) in the current study which was less as compared to the other study published from Mumbai [22]. Notably, we could diagnose tuberculosis and rifampicin resistance within four hours, which otherwise could have taken four days to four weeks. Moreover, ATT was started on day two in patients who were Xpert® MTB/RIF-positive as compared to those who were negative as they received it on day seven (median duration seven days). As shown previously, delayed diagnosis and the start of treatment worsens the prognosis and increases the mortality of PTB patients [23]. Thus, Xpert® MTB/RIF assay is clinically vital and useful.

Nevertheless, the present study has some limitations. It is a retrospective study, and hence, we did not compare Xpert® MTB/RIF assay with respect to the culture owing to its variable yield in PTB. Also, the sample size of the current study was small. Additionally, a cost-effective analysis for Xpert® MTB/RIF assay could not be performed.

## Conclusions

Xpert® MTB/RIF assay on peritoneal tissue obtained by peritoneoscopy has fair sensitivity and excellent specificity with a distinct advantage in the early diagnosis of MDR tuberculosis.
